# Customer–Company Identification as the Enabler of Customer Voice Behavior: How Does It Happen?

**DOI:** 10.3389/fpsyg.2020.00777

**Published:** 2020-04-22

**Authors:** Yang Ran, Hao Zhou

**Affiliations:** School of Business, Sichuan University, Chengdu, China

**Keywords:** customer–company identification, customer voice behavior, felt responsibility for constructive change, self-impact, organizational identity theory

## Abstract

In this study, we define customer voice behavior as a consumer’s extra-role communicative behavior of offering suggestions or opinions to enterprises. We classified customer voice behavior into two dimensions: promotive and prohibitive voices. Furthermore, we explored the relationship between customer–company (C–C) identification and customer voice behavior. From a sample of 394 university students who were customers of food delivery services, the results showed that C–C identification was positively related to both promotive and prohibitive voices while felt responsibility for constructive change (FRCC) played a mediating role between C–C identification and both kinds of customer voice behavior. In addition, we found the moderating effects of self-impact, which could strengthen the impacts of FRCC on customer voice behavior and the mediating effects of FRCC. The theoretical and practical implications of this study are also discussed.

## Introduction

As today’s business environment becomes more and more intense, customer participation plays a critical role in product development and the improvement of a firm’s performance (Bonner, 2010; Lin and Huang, 2013; Wang et al., 2019). Consumers are regarded as “partial employees” who participate in the production or service operations of companies (Bowen et al., 2000; Groth, 2005) while customer voice can be an important method for helping them assume this new role. Customer voice is proactive behavior which can bring about positive improvement or change to an existing state (Bashshur and Oc, 2014; Min and Kim, 2019). We suggest that it deserves more attention, especially in the current era.

On the one hand, previous studies have demonstrated the numerous benefits, such as increased customer loyalty (Bhodgett et al., 1995; Bettencourt, 1997), improved offerings and the prevention of future problems (Ahmad, 2002; Jain et al., 2003; Assaf et al., 2015), opportunities to correct errors (Priluck, 2003), and the maintenance of customer-firm relationships (Lacey, 2012; Béal and Sabadie, 2018), of direct face-to-face customer voice to organizations. On the other hand, under the high-speed development of Internet, ordinary consumers could conveniently express their voice through many online channels, such as social media, as well as brand community and shopping platforms (Stevens et al., 2018). Indirect online customer voice is also beneficial to a firm’s products and services (Jin et al., 2019; Yang et al., 2019). Moreover, many companies have recognized the importance of customer voice and are paying more attention to it. For example, Starbucks established an online crowdsourcing platform named “My Starbucks Idea” to collect examples, such as ideas and suggestions, of customer voice and obtain valuable ideas for developing new drinks, improving service, and promoting the overall performance of the company (Hossain and Islam, 2015).

Reviewing the extant literature on customer voice behavior, we found some limitations and gaps in the relevant research. First, several scholars who study consumer behavior have referred to customer voice as mainly a type of complaint behavior (Boote, 1998) that vocally expresses complaints to the service provider (Marquis and Filiatrault, 2002; Bove and Robertson, 2005; Crutchfield, 2008). However, this definition of customer voice is not suitable for current research. Scholars are finding that consumers can express more to service providers by the use of their voice, which includes suggestions, satisfaction, compliments, and word of mouth (Assaf et al., 2015; Ma et al., 2015; Cossío-Silva et al., 2016; Béal and Sabadie, 2018; Zhang, 2019). There is a lack of conceptual definitions of customer voice applicable to the current situation.

Second, although previous scholars have spent some effort on the dimensions of customer voice, the classification of this construct needs to be improved. For example, Assaf et al. (2015) suggested that customer satisfaction and complaints were two essential variables of customer voice, whereas Béal and Sabadie (2018) divided customer voice into two dimensions such as complaint intentions and suggestions for service improvement. These classifications cannot include all the above-mentioned forms (e.g., compliments and word of mouth) of customer voice, which demands an optimization.

Third, existing studies focus on the impacts of different forms of customer voice on enterprises while the antecedents and mechanisms of customer voice are scanty. Bove and Robertson (2005) proposed that trust and commitment could be predictors of customer voice while Béal and Sabadie (2018) found that psychological ownership could also stimulate customer voice behavior, which has been shown to offer positive contributions to enterprises. Hence, more effort is required for the exploration of the antecedents of customer voice (Bove and Robertson, 2005; Béal and Sabadie, 2018).

To fill the research gap in customer voice behavior, this article proposes a new definition that refers to the concept of employee voice (Van Dyne and LePine, 1998). Specifically, we refer to customer voice as the extra-role communicative behavior by which customers actively offer suggestions or opinions to improve an enterprise’s status. Drawing upon Liang et al.’s (2012) classification of employee voice behavior, we classified customer voice behavior into two dimensions: promotive and prohibitive. Promotive voice indicates the innovative ideas and suggestions offered by a customer for the improvement of an enterprise’s efficiency while prohibitive voice refers to the expression of opinions on practical and potential problems existing with an enterprise’s product, service, or management that harm the enterprise or customer. Despite the numerous benefits of customer voice behavior, when the risks and potential losses resulting from voice behavior are considered, not all consumers would like to offer their suggestions or opinions to the service providers (Krefting and Powers, 1998; Bove and Robertson, 2005).

On the basis of the theory of organizational identity (Ashforth and Mael, 1989), we propose that customer–company (C–C) identification could be an important predictor of customer voice behavior. C–C identification refers to the feeling of an individual’s belongingness to an enterprise, which has always been a central construct in the field of relationship marketing (Bhattacharya and Sen, 2003). C–C identification is also a crucial reason for why a customer is willing to maintain close relationships with and offer support to an enterprise (Su et al., 2016). The stronger the customers’ sense of C–C identification, the stronger would be the supportive behavior (Ahearne et al., 2005). Therefore, we posited that C–C identification could be an enabler of customer voice behavior.

According to organizational identity theory (Ashforth and Mael, 1989), when individuals identify with an organization, they feel a rational sense of responsibility that influences them to take actions, such as voice behavior, to do help for the organizational performance (Fuller et al., 2006). Thus, we propose that felt responsibility for constructive change (FRCC) plays a mediating role between C–C identification and customer voice. For this study, we explored the moderating role of self-impact, which variable reflects the feeling individuals experience when they are able to control important results and consequences (Spreitzer, 1995). Based on expectancy theory, individuals decide whether or not to engage in a particular action according to the actions’ expected outcome (Vroom, 1964). Individuals with higher self-impact have more positive expectations of behavioral outcomes, so they are more motivated to engage in beneficial behavior toward an organization (Wang et al., 2015). Therefore, we propose that a sense of self-impact could moderate the effects of C–C identification on customer voice behavior.

In general, we proposed a moderated mediation model to figure out the intrinsic mechanism and boundary conditions by which C–C identification affects customer voice. This article contributes to the extant literature in several ways. First, drawing upon the concept of employee voice behavior, we offer a new definition of customer voice and classify it into promotive and prohibitive voices (Van Dyne and LePine, 1998; Liang et al., 2012). Further, this study is the first to have empirically tested an integrated conceptual model of how and when C–C identification influences customer voice behavior.

## Literature Review and Hypotheses

In this section, we review the relevant concepts such as customer voice behavior, C–C identification, FRCC and self-impact. Based on the organizational identity theory and expectancy theory, we propose the hypotheses and theoretical model.

### Customer Voice Behavior

Hirschman (1970) first proposed the construct of voice behavior and put forward that employees usually respond to low job satisfaction in two ways: exit or voice. Employees with high loyalty prefer to speak out rather than leave their firms. Hirschman’s opinion has been developed by many other scholars into several directions, such as the framework of exit, voice, loyalty, and neglect (Rusbult et al., 1982; Dan, 1983; Rusbult et al., 1988; Drigotas et al., 1995). However, some scholars believe that “dissatisfaction with the organization” as the premise of voice behavior is an inappropriate connotation because employees who are satisfied with their organizations could also exhibit voice behavior for the purpose of improving organizational effectiveness. Van Dyne and LePine (1998) referred employee voice as a type of employee’s extra-role communicative behavior of offering constructive suggestions to improve an organization. This definition of employee voice behavior from the perspective of “extra-role behavior” has been generally recognized by scholars (Zhou and George, 2001; Avery and Quinones, 2002). According to this definition, Liang et al. (2012) divided employee voice behavior into two dimensions, which are promotive and prohibitive voices, that are already being applied in current research (Chamberlin et al., 2017; Arain et al., 2019). Promotive voice refers mainly to ideas or suggestions which could do help to organizational efficiency while prohibitive voice involves the expression of opinions on issues that are not conducive to organizational development.

In the literature on consumer behavior, customer voice was originally regarded as a type of customer complaint behavior (Boote, 1998), which meant a customer’s complaining to service providers about problems with the latter’s services. Such behavior would be an expression of customer dissatisfaction (Marquis and Filiatrault, 2002; Crutchfield, 2008; Lacey, 2012). With the development of research into customer voice, scholars have proposed that customer voice is not merely about customer complaints when customers are dissatisfied but also about making suggestions to enterprises via voice (Bettencourt, 1997; Cossío-Silva et al., 2016). Suggestions could help enterprises improve their service quality and efficiency, and so, could also be regarded as a form of customer voice (Béal and Sabadie, 2018). In addition, satisfaction, compliments, and word of mouth expressed by customers in contact with enterprises are similarly regarded as part of customer voice behavior (Assaf et al., 2015; Ma et al., 2015; Zhang, 2019).

As the fast development of network, ordinary consumers could express their voice through online platforms, including social media and online shopping platforms (Stevens et al., 2018). Some scholars have focused on different forms of expression of voice, such as electronic word of mouth (Zhang, 2019), compliments and complaints on social media (Ma et al., 2015). Moreover, there is little research on the classification of customer voice. For example, Assaf et al. (2015) suggested that customer satisfaction and complaints were two essential customer voice variables, whereas Béal and Sabadie (2018) divided customer voice into two dimensions such as complaint intentions and suggestions for service improvement.

A summary of the existing literature leads to the conclusion that the concept of customer voice could be founded on the similar concept of employee voice, which originated in the field of organizational behavior. Such a notion has attracted much attention in the process of construct development. Although researchers are constantly studying the positive impacts of various forms of customer voice on enterprises, there is no conceptual definition of customer voice for the current situation and its dimensions need further improvement. To make up for these gaps by referring to Van Dyne and LePine’s (1998) definition of employee voice, this article defines customer voice behavior as the extra-role communicative behavior in which customers actively offer suggestions or opinions to improve an enterprise’s product or service status. At the same time, consumers are considered as “partial employees” who act as virtual members of enterprises. Drawing on Liang et al.’s (2012) classification about employee voice behavior, we divided customer voice behavior into promotive and prohibitive voices, of which the former refers to customer voice intended to improve the efficiency of enterprises by offering innovative ideas and suggestions while the latter refers to informing enterprises of problems that may harm the enterprises themselves and their customers.

In general, we conceptualized the notion of customer voice for the following reasons. Firstly, the view that customer voice is an extra-role behavior of consumers has become a consensus among scholars (Spake et al., 2003; Bove and Robertson, 2005). Regardless of the form taken by customer voice, it is always beneficial to the enterprise. Therefore, we believe that customer voice is essentially a kind of consumer behavior that is exhibited in the offering of opinions and suggestions for improving the current states of products and services. Secondly, starting from its content, we classified customer voice into promotive and prohibitive voices. This classification applies appropriately to the existing research on customer voice. Finally, customer voice could be based on the similar concept of employee voice by considering consumers as “partial employees” who act as virtual members of an enterprise. Therefore, basing the definition and classification of customer voice behavior on employee voice behavior is reasonable.

### Customer–Company (C–C) Identification and Customer Voice Behavior

Bove and Robertson (2005) proposed that relationship variables would be important antecedents for customer voice behavior. Utilizing this idea, this study focused on C–C identification. Dutton et al. (1994) proposed that the identification of a person to an organization comes from the similarity between the image of the organization and the self-concept of the individual. The strong sense of common identity makes the individual regard the organization as part of themselves; hence, they would think that doing something beneficial for the organization would be the same as doing something beneficial for themselves. Bhattacharya and Sen (2003) initially proposed the concept of C–C identification on the foundation of organizational identification and defined the former as the close relationship that a customer feels between an enterprise and themselves. From such a relationship, consumers could satisfy their psychological needs for self-identification and affect the consumptive behavior. The authors believe that the stronger the customer’s sense of C–C identification, the more supportive behavior they would exhibit toward an enterprise. Albert et al. (2000) also pointed out that a customer with a high level of identification was more likely to regard an enterprise and themselves as connected, so their attitudes would be more similar to those of the enterprises. Such a customer would be more concerned with the future development of the enterprise. Therefore, a customer with a high level of C–C identification would be more motivated to exhibit voluntary positive behaviors toward the enterprise.

Research on C–C identification is supportive of the view that C–C identification has an active impact on extra-role consumer behaviors (Ahearne et al., 2005; Wu and Tsai, 2007; Karaosmanoglu et al., 2011). Ahearne et al. (2005) explored the outcome variables of C–C identification empirically by collecting data from the pharmaceutical industry. Their results showed that when physicians (customers) identify with enterprises, purchasing the products of the enterprises could be a means of self-expression. In addition, the physicians (customers) who identify with the enterprises exhibited extra-role behaviors, such as recommending products to others and offering suggestions to the enterprises. By such actions, they exhibit their support of the enterprises. Another empirical study by Wu and Tsai (2007) concluded that improving C–C identification could promote customers’ offers of positive complaint and suggestions, which two types of behaviors are similar to the customer prohibitive and promotive voice behaviors described in this article. With the prevalence of customer citizenship behavior (CCB), many studies have shown that C–C identification is a key factor in the stimulation of CCB. For instance, Hur et al. (2018) pointed out that higher levels of customer identification with enterprises (e.g., banks) resulted in a greater willingness on the part of the customers to engage in citizenship behaviors, such as assisting bank employees and recommending services to other people. Wu et al. (2017) demonstrated that identification had a positive impact on CCB on social networking sites.

The abovementioned views suggest that, as an extra-role behavior of CCB (Groth, 2005; Bove et al., 2009), customer voice should also be affected by C–C identification. Accordingly, this study predicted:

H1. C–C identification is positively related to promotive (H1a) and prohibitive (H1b) voice behaviors.

### The Mediating Role of Felt Responsibility for Constructive Change (FRCC)

According to the theory of organizational identity (Ashforth and Mael, 1989), when an individual identifies with an organization, they consider themselves to be among its members and feel a rational sense of responsibility, out of which they would take action to help the organization improve its performance. Following this rationale, we propose that FRCC is a mediating mechanism that explains the impacts of C–C identification on customer voice behaviors.

Felt responsibility for constructive change FRCC is a critical psychological state in which individuals feel accountable and responsible for their work (Hackman and Oldham, 1976). Morrison and Phelps (1999) defined it as an individual’s belief in a personal obligation to bring about constructive change. Campbell (2017) pointed out that an enhanced personal sense of identification, i.e., a strong sense of ownership toward the organization, should underlie feelings of responsibility and a willingness to make an exceptional effort for the organization. When individuals feel a sense of responsibility for their organizations, they are more likely to exhibit extra-role behaviors (Pearce and Gregersen, 1991). Previous studies have shown that FRCC had positive impacts on employee voice behaviors (Fuller et al., 2006), including promotive and prohibitive voices (Chamberlin et al., 2017; Arain et al., 2019). Similarly, we predict that, when a customer acts as a partial employee and feels a sense of responsibility for an enterprise, they would also want to help through positive extra-role behaviors, such as promotive and prohibitive voices.

In conclusion, a customer’s identification with an organization would motivate their FRCC, which, in turn, would encourage them to express responsibility through supportive behaviors. The customer would essentially be acting as a partial employee of the organization. With this view in mind, we proposed the following:

H2. FRCC mediates the positive relationship between C–C identification and both promotive (H2a) and prohibitive (H2b) voice behaviors.

### The Moderating Role of Self-Impact

This study further explores the boundary conditions of the effects of C–C identification on customer voice behaviors. We chose the individual factor as the moderator and focused on self-impact. Ashforth and Mael (1989) proposed that self-impact should refer to the degree to which an individual influences strategy and performance at work. Spreitzer (1995) established a model of psychological empowerment, which regards impact as a sub-dimension of psychological empowerment. Spreitzer (1995) defined self-impact as an individual’s feeling of being able to control important results and consequences in their organization. According to this definition, we can infer that self-impact is a variable regarding personal perception of their own abilities. In the context of marketing, we define customer self-impact as a customer’s own perception of their control and influence on their social circle.

According to expectancy theory, an individual decides whether or not to engage in a particular action according to an expected outcome (Vroom, 1964). Employees with high self-impact perceive a stronger link between their behavior and work outcomes while believing that they are able to solve organizational problems independently. High self-impact can promote an individual’s expectations of behavioral outcomes and motivate them to engage in positive behavior toward an organization (Wang et al., 2015). Also, Tangirala and Ramanujam (2012) confirmed that an employee’s self-impact could promote voice behavior.

According to organizational identity and expectancy theories (Vroom, 1964; Ashforth and Mael, 1989), customers wish to help and improve the performance of the enterprises with which they identify. Customers with high self-impact perceive their behaviors as being able to affect more people and expect their behavioral outcomes to have better effects. Hence, such customers would feel more motivated to participate in citizenship behavior in favor of the enterprises, and, in this way, satisfy their psychological needs for self-identification. In contrast, customers with low self-impact would not be sufficiently motivated to exhibit extra-role behaviors. Therefore, this study predicted:

H3. Self-impact positively moderates the effect of FRCC on both promotive (H3a) and prohibitive (H3b) voice behaviors.

From the above hypotheses, we further inferred that the mediating role of FRCC between C–C identification and customer voice behavior may also be moderated by self-impact. Because the indirect effect of C–C identification on customer voice behavior is mediated by FRCC, the relationship between FRCC and customer voice would be positively moderated by self-impact. Therefore, when the level of self-impact is high, the indirect effect of C–C identification on customer voice would be enhanced. Hence, we predicted:

H4. Through FRCC, self-impact positively moderates the effects of the mediated relationship between C–C identification and both promotive (H4a) and prohibitive (H4b) voice behaviors.

**FIGURE 1:**
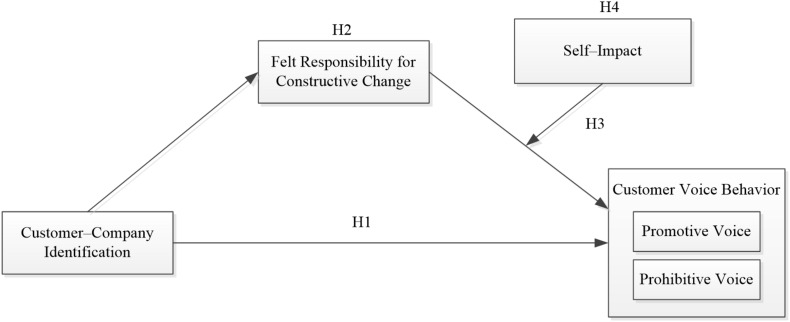
Theoretical model.

The theoretical model of this research is shown in Figure 1.

## Methodology

In this section, we first introduce the background and participants of our research. Then we introduce the measurements of main constructs and present sample items of each variable. Finally, we summarize the statistical methods used in our study.

### Research Background and Participants

The Chinese food delivery industry has been developing very rapidly in recent years. According to an industry market report (iiMedia, 2018), the number of customers exceeded 350 million people in 2018 and the market has maintained double-digit expansion every year. Without needing to leave their homes, customers can use mobile apps to choose and order the products (meals and beverages), which are then delivered to their homes. This convenient service is becoming increasingly popular with the public.

University students are an important target market for the food delivery industry, which has active consumer groups and huge growth potential (iiMedia, 2018). We chose this target market as the focus and distributed 536 questionnaires to undergraduate students at a university in southwestern China. We received 394 valid responses. The final sample consisted of 229 females and 165 males, of which 123 (31.2%) were first-year, 135 (34.3%) were second-year, 74 (18.8%) were third-year, and 62 (15.7%) were fourth-year students. On the frequency of their usage of food delivery services, 295 (74.9%) respondents replied that they used such services three times a week while the remaining 99 (25.1%) used the services at least four times a week.

### Measurements

University students may be customers of multiple food delivery companies. We asked participants to choose one as the evaluation target, usually the one they used most. To measure C–C identification, this study used the measurement developed by Bergami and Bagozzi (2000). They provided a chain of Venn diagrams showing the degree of overlap between customers and enterprises from low to high. The respondents chose the degree of overlap that represented their relationship with the company.

The FRCC was assessed by a five-item scale developed by Morrison and Phelps (1999). We adjusted the items to fit the perspective of the customer. A sample item here was: “I feel much obligation to help the [company name] improve its *status quo*”. Cronbach’s alpha was 0.930 for this survey.

Self-impact was assessed by a three-item scale developed by Spreitzer (1995). We adjusted the items to fit the perspective of the customer. A sample item here was: “I have significant influence over what happens in my social circle”. Cronbach’s alpha was 0.938 for this survey.

Customer voice behavior was adapted from Liang et al.’s (2012) ten-item scale, which measured employee promotive and prohibitive voices. Following our definition of customer voice behavior, we adapted this scale into a two-dimensional scale of six items for customer voice behavior. A sample item here for customer promotive voice was: “I would proactively suggest new projects which are beneficial to the [company name]”. A sample item here for customer prohibitive voice was: “I would reflect the possible problems to the [company name] in its product and service”. Cronbach’s alphas were 0.952 and 0.916 for customer promotive and prohibitive voices, respectively.

We followed the procedure of translation and back-translation suggested by Brislin (1980) to ensure translation equivalence. Seven-point Likert scales (1: “strongly disagree” to 7: “strongly agree”) were used for all the observed variables, except C–C identification. Also, we set the participants’ genders and grades as control variables. All the detailed scales are shown in Appendix.

### Statistical Analysis

To establish the validity of the research constructs, a confirmatory factor analysis (CFA) was performed through AMOS software. We probed and contrasted a five-factor model with two alternative models. Furthermore, we also performed a comparison of the five-factor model and a single-factor model to evaluate the risk for common method biases (Podsakoff et al., 2003).

To test the proposed hypotheses regarding direct relationships among variables, Pearson’s bivariate correlation was used as a preliminary test. Then we used the SPSS macro PROCESS developed by Hayes (2013) as a robust test for testing these hypotheses. First, we conducted multiple regression analysis using PROCESS template 4 to test the direct and indirect effect of C–C identification on customer voice behavior. Second, to assess the complete moderated mediation model, template 14 was specified in the PROCESS. In addition, we noted that the demographic factor such as gender and grade could influence the research variables. Therefore, we included these variables as controls in our hypothesis test.

## Results

In this section, we present the results of measurement validation, correlation analysis and hypotheses testing. In addition, we use some charts to show these analysis results more intuitively.

### Measurement Validation

Confirmatory factor analysis was used to assess discriminant validity with Amos 23.0. As shown in Table 1, the five-factor model provided a good model fit (χ^2^ = 331.77, df = 81, CFI = 0.96, TLI = 0.94, and RMSEA = 0.08), whereas the four-factor model (promotive and prohibitive voices combined) and three-factor model (promotive voice, prohibitive voice, and FRCC combined) fitted poorly.

**TABLE 1:** Results of CFA.

Model	χ^2^	*df*	χ^2^/*df*	CFI	TLI	RMSEA
Five-factor model	331.77	81	4.09	0.96	0.94	0.08
Four-factor model	572.61	85	6.74	0.92	0.90	0.12
Three-factor model	1146.39	88	13.03	0.82	0.78	0.17
Single-factor model	2153.34	91	23.66	0.64	0.59	0.24

*Five-factor model: C–C identification, FRCC, self-impact, promotive voice, and prohibitive voice; four-factor model: promotive and prohibitive voice were combined; three-factor model: promotive voice, prohibitive voice, and FRCC were combined; single-factor model: all five factors were combined.*

Because all the items had been self-reported, the problem of common method biases may exist. Following the suggestion of Podsakoff et al. (2003), we performed a single-factor test in the CFA. As shown in Table 1, the results produced a poor model fit, which indicated that there were no significant common method biases in our measurement.

Table 2 contains the means, standard deviations, and correlation matrix of each construct. C–C identification was positively related to promotive voice (*r* = 0.305, *p* < 0.01), prohibitive voice (*r* = 0.332, *p* < 0.01), and FRCC (*r* = 0.339, *p* < 0.01). Also, there were significant positive correlations between FRCC and both promotive voice (*r* = 0.749, *p* < 0.01) and prohibitive voice (*r* = 0.653, *p* < 0.01). These results provided preliminary support for our hypotheses.

**TABLE 2:** Means, standard deviations, and correlations.

Variable	*M*	SD	1	2	3	4	5	6	7
1. Gender	1.58	0.494	–						
2. Grade	2.19	1.047	0.012	–					
3. C–C identification	3.21	1.566	0.000	0.061	–				
4. FRCC	4.47	1.275	0.146**	–0.051	0.339**	0.930			
5. Self-impact	4.35	1.271	–0.062	–0.093	0.246**	0.411**	0.938		
6. Promotive voice	4.46	1.295	0.147**	−0.111*	0.305**	0.749**	0.446**	0.952	
7. Prohibitive voice	4.72	1.219	0.191**	−0.106*	0.332**	0.653**	0.408**	0.785**	0.916

N = 394. Internal consistency reliabilities in the diagonal. Gender, 1 = male, 2 = female. Grade, 1 = first-year students, 2 = second-year students, 3 = third-year students, and 4 = fourth-year students. *p < 0.05, **p < 0.01 (two tailed).

### Hypotheses Testing

We used a series of regression analysis to test our hypotheses. As shown in Table 3, after the effects of gender and grade had been controlled, C–C identification emerged as positively related to both promotive voice (*B* = 0.26, SE = 0.04, *p* < 0.01, Model 2) and prohibitive voice (*B* = 0.26, SE = 0.04, *p* < 0.01, Model 5), thereby supporting Hypotheses 1a and 1b.

**TABLE 3:** Results of regression analysis.

	FRCC	Customer promotive voice			Customer prohibitive voice		
			
	Model 1	Model 2	Model 3	Model 4	Model 5	Model 6	Model 7
Predictor variable	*B* (SE)	*B* (SE)	*B* (SE)	*B* (SE)	*B* (SE)	*B* (SE)	*B* (SE)
Gender	0.38(0.12)**	0.46(0.12)**	0.19(0.09)*	0.24(0.09)**	0.48(0.11)**	0.26(09)**	0.32(0.09)**
Grade	−0.09(0.06)	−0.16(0.06)**	−0.10(0.04)*	−0.08(0.04)	−0.15(0.05)**	−0.10(0.04)*	−0.08(0.04)
C–C identification	0.28(0.04)**	0.26(0.04)**	0.06(0.03)	0.03(0.03)	0.26(0.04)**	0.11(0.03)**	0.09(0.03)**
FRCC			0.72(0.04)**	0.40(0.09)**		0.56(0.04)**	0.24(0.10)*
Self-impact				−0.11(0.10)			−0.13(0.11)
FRCC × self-impact				0.06(0.02)**			0.06(0.02)**
*R*^2^	0.14	0.14	0.58	0.61	0.16	0.46	0.49
Δ*R*^2^			0.44	0.03		0.30	0.03
*F*	21.53**	21.24**	131.56**	99.59**	25.34**	82.10**	62.13**

*B* = unstandardized regression coefficients. *N* = 394. Bootstrap sample size = 5000. *p < 0.05; **p < 0.01.

As shown in Table 3, C–C identification was found to be positively related to FRCC (*B* = 0.28, SE = 0.04, *p* < 0.01, Model 1). Furthermore, FRCC was positively related to promotive voice (*B* = 0.72, SE = 0.04, *p* < 0.01, Model 3), whereas C–C identification had no significant effect (*B* = 0.06, SE = 0.03, *p* > 0.05, Model 3). In addition, FRCC was positively related to prohibitive voice (*B* = 0.56, SE = 0.04, *p* < 0.01, Model 6). Although the effect of C–C identification was significant, it was obviously smaller (*B* = 0.11, SE = 0.03, *p* < 0.01, Model 6). We further tested the two mediating effects using 5000 bootstrapping samples. The analyses indicated a significant mediating effect between C–C identification and promotive voice via FRCC [*B* = 0.12, SE = 0.04, 95% CI (0.05, 0.19), excluding zero]. Similarly, the mediating effect for prohibitive voice was significant [*B* = 0.11, SE = 0.03, 95% CI (0.05, 0.17), excluding zero]. Thus, Hypotheses 2a and 2b were supported.

**FIGURE 2:**
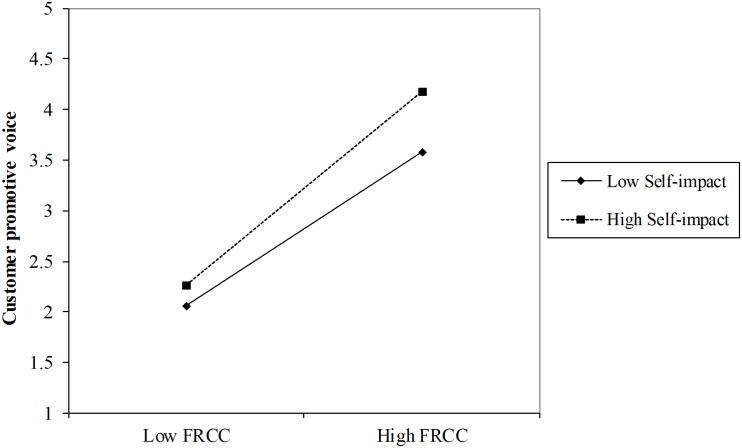
Moderating effect of self-impact on the relationship between FRCC and customer promotive voice.

**FIGURE 3:**
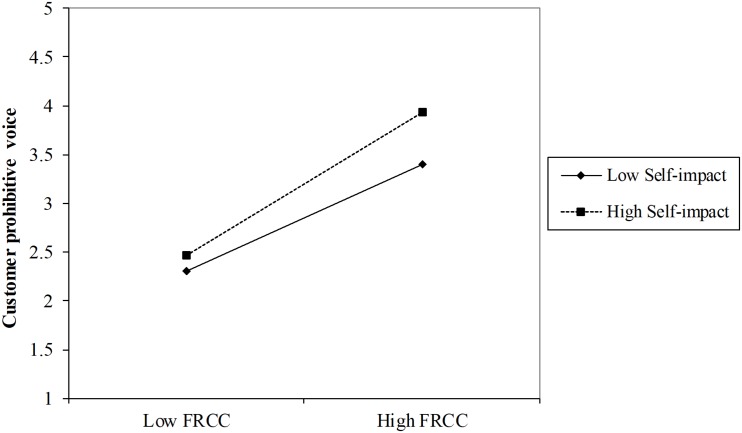
Moderating effect of self-impact on the relationship between FRCC and customer prohibitive voice.

Hypothesis 3 proposes the moderating effect of self-impact on the relationship between FRCC and customer voice behavior. Table 3 shows that the interaction between FRCC and self-impact had a significant effect on both promotive voice (*B* = 0.06, SE = 0.02, *p* < 0.01, Model 4) and prohibitive voice (*B* = 0.06, SE = 0.02, *p* < 0.01, Model 7). Thus, Hypotheses 3a and 3b were supported. As shown in Figures 2, 3, the positive impacts of FRCC on both promotive and prohibitive voice were stronger for those respondents with high self-impact.

Hypothesis 4 states that the mediating effect of C–C identification on customer voice through FRCC would be positively moderated by self-impact. We used the SPSS macro PROCESS developed by Hayes (2013) to test the moderated mediation effect. The bootstrap sample size was set to 5000. The results showed that, for customer promotive voice, the index of moderated mediation was 0.02 [95% CI (0.00, 0.03), excluding zero], so Hypothesis 4a was supported. For prohibitive voice, the index of moderated mediation was 0.02 [95% CI (−0.00, 0.04), including zero], so Hypothesis 4b was not supported.

## Discussion

In this section, we summarize the general results, the theoretical implications and managerial implications of this study. Besides, we introduce the limitations and future directions at the end of this section.

### General Results

This study proposed a moderated mediation model to explore the underlying mechanism between C–C identification and customer voice behavior on the basis of organizational identity and expectancy theories. The results showed that C–C identification was positively related to customer voice behavior and FRCC played a mediating role between them. Since customers were considered as “partial employees” of organizations, the positive correlation between C–C identification and customer voice also indirectly validated the views of other researchers. For instance, some scholars of organizational behavior have found employee identification to be positively related to employee voice behavior (Liu et al., 2010; Yang and Liu, 2014). Meanwhile, FRCC’s positive relationship to both promotive and prohibitive voices also validated the opinions of Chamberlin et al. (2017) and Arain et al. (2019). Furthermore, we tested the moderating effect of customers’ self-impact and found a positive moderating effect on the second stage (FRCC to customer voice behavior) of the concept model.

The results of the mediation analysis demonstrated that FRCC has a fully mediating effect on the relationship between C–C identification and customer promotive voice but a partially mediating effect on the relationship with prohibitive voice. This difference may be caused by the different meanings of the two types of customer voice behavior, as other studies are consistent in indicating that prohibitive voice implies a higher risk to the customer (Chamberlin et al., 2017; Arain et al., 2019). Customers may worry about potential losses when they display prohibitive voice. Therefore, their sense of responsibility is not sufficient to motivate them to exhibit prohibitive voice.

There may be other variables that mediate the relationship between C–C identification and promotive voice behavior. At the same time, the results demonstrated that self-impact positively moderates the indirect effect of C–C identification on promotive voice through FRCC, but the moderated mediation effect on prohibitive voice is not significant. The possibility of other mediating variables between C–C identification and customer promotive voice behavior was also demonstrated.

### Theoretical and Managerial Implications

These findings contribute to the present literature in at least three ways. First, a new definition of customer voice was formulated from the perspective of positive extra-role behavior, which is different from the definitions of previous studies that had referred to customer voice as the complaint behaviors of customers who feel dissatisfaction. In this article, customer voice was defined as the extra-role communicative behavior of customers who actively offer suggestions or opinions to improve an enterprise’s products and services. On the basis of the content of customer voice, we classified it into promotive and prohibitive voices. The new definition and dimensions of customer voice proposed in this article will contribute to the existing research on customer voice behavior and offer implications to future researchers.

Second, CCB has been the focus of many scholars in recent years, but they have mainly concentrated on providing feedback, helping other customers, and other behaviors. As customer voice behavior is an important type of CCB, the pertinent research needs to be further explored. This study’s focus on customer voice behavior enriches the current investigations of CCB. Furthermore, we explored the underlying mechanism between C–C identification and customer voice on the basis of organizational identity theory. The results indicated that C–C identification could be an antecedent of customer voice behavior and the FRCC could play a mediating role between them. These findings may shed light on future research into CCB.

Third, every customer is a unique individual, and the differences among customers is a core point that enterprises should consider when planning marketing strategies. This study chose self-impact as a moderator, which reflects an individual’s ability to explore the behavioral differences of consumers at different levels of self-impact. The results of data analysis confirmed that customers with higher personal self-impact would be more motivated to exhibit positive voice behavior.

The findings of our research also provide some valuable practical implications for enterprises. Knowledge of customer voice behavior could help firms find any problems with their products and services, as well as encourage consumers to submit useful suggestions. The good use of customer voice could prove beneficial to enterprises and improve their competitiveness. Therefore, this study provides a new method by which enterprises can improve their advantages by not relying solely on their internal resources but also making use of external resources, such as customers, to improve their performance.

The results also indicate that the higher the C–C identification, the more likely customers are to engage in positive voice behavior, which can have implications for the marketing activities of enterprises. For instance, enterprises often need consumers to undertake market tests when developing new products. However, collecting feedback from consumers often requires plenty of resources and costs. In such situations, enterprises can target customers with high C–C identification, because such customers are not only willing to participate in these activities but also more likely to offer reliable and useful suggestions. In this way, enterprises can reduce their costs and improve their efficiency.

With an understanding of the role of FRCC in mediating between C–C identification and customer voice behavior, enterprises can promote customer voice behavior by stimulating and enhancing their customers’ feelings of responsibility. For example, enterprises can publicize the adoption of their customers’ ideas or suggestions and donate money to public welfare funds. By stimulating their customers’ sense of responsibility, enterprises can hope to receive a higher number of suggestions.

## Limitations and Future Directions

There are some limitations to this study that could provide opportunities for future studies. Firstly, we used cross-sectional data to test the theoretical model, but longitudinal data would be better for future studies.

Secondly, we adapted the definition of customer voice behavior from that of employee voice behavior and divided the former into two dimensions (promotive and prohibitive voices). The validity of this classification and its applicability to other situations require further examination. Although some scholars regard consumers as “partial employees” for organizations, the relationship between consumers and enterprises is not the same as that between employees and organizations. The employees are subordinate to the enterprises, but the customers are more like partners with enterprises. Thus, the customer voice scale adapted from the employee voice we used may not completely capture the nature of customer voice behavior, and the development of a suitable customer voice scale could be a direction for future studies.

Thirdly, we found only a few studies on customer voice behavior in the existing literature, as well as its underlying mechanisms and boundary conditions. Customer voice behavior can bring many benefits to enterprises. A more in-depth focus on this concept would be beneficial, so we would encourage more research into the underlying mechanisms of customer voice behavior from different theoretical perspectives and into the personal ability moderators of customer voice behavior.

Fourthly, in order to test the theoretical model, we chose the Chinese food delivery industry as the research context and selected university students as participants, which may cause some limitations. Future studies could consider collecting data from more groups of consumers. Besides, the theoretical model we proposed need to be further verified under other industries and contexts.

## Data Availability Statement

The datasets generated for this study are available on request to the corresponding author.

## Ethics Statement

Ethical review and approval was not required for the study on human participants in accordance with the local legislation and institutional requirements. Written informed consent from the participants was not required to participate in this study in accordance with the national legislation and the institutional requirements.

## Author Contributions

YR: conceptualization, methodology, software, formal analysis, and original draft preparation. HZ: writing, reviewing, editing of the manuscript, project administration, and funding acquisition.

## Conflict of Interest

The authors declare that the research was conducted in the absence of any commercial or financial relationships that could be construed as a potential conflict of interest.

## Appendix

### Measures of Core Constructs

#### Customer-Company Identification

We sometimes strongly identify with a company. This occurs when we perceive a great amount of overlap between our ideas about who we are as a person and what we stand for (i.e., our self-image) and of who this company is and what it stands for (i.e., the company’s image).

Imagine that the circle at the left in each row represents your own personal identity and the other circle, at the right, represents the company’s identity. Please indicate which case (A, B, C, D, E, F, G, or H) best describes the level of overlap between your and the company’s identities. (Circle appropriate letter).

#### Felt Responsibility for Constructive Change

1. I feel a personal sense of responsibility to bring about change.
2. It’s up to me to bring about improvement for the [company name].
3. I feel obligated to try to introduce the [company name] where appropriate.
4. Correcting problems for the [company name] is my responsibility.
5. I feel much obligation to help the [company name] improve its *status quo*.

#### Self-Impact

1. My impact on what happens in my social circle is large.
2. I have a great deal of control over what happens in my social circle.
3. I have significant influence over what happens in my social circle.

#### Promotive Voice

1. I would proactively suggest new projects which are beneficial to the [company name].
2. I would raise suggestions to improve the [company name]’s service procedure.
3. I would make constructive suggestions to improve the [company name]’s operation.

#### Prohibitive Voice

1. I would reflect the possible problems to the [company name] in its product and service.
2. I would report to the [company name] the problems encountered in receiving its service.
3. I would comment on issues that are not conducive to the development of [company name].
